# Enhanced Stature in the Elderly: The Immediate Impact of Acute Postural Exercises

**DOI:** 10.3390/sports12030085

**Published:** 2024-03-20

**Authors:** Arnulfo Ramos-Jiménez, Ismael Antonio García-Hernández, Isaac Armando Chávez-Guevara, Rosa Patricia Hernández-Torres, Miguel Murguía-Romero, José Miguel Martínez-Sanz, Marco Antonio Hernández-Lepe

**Affiliations:** 1 Department of Health Sciences, Biomedical Sciences Institute, Autonomous University of Ciudad Juarez, Chihuahua 32310, Mexico; aramos@uacj.mx (A.R.-J.); al216946@alumnos.uacj.mx (I.A.G.-H.); 2 Conahcyt National Laboratory of Body Composition and Energetic Metabolism (LaNCoCoME), Tijuana 22390, Mexico; isaac.chavez@uacj.mx (I.A.C.-G.); rphernant@gmail.com (R.P.H.-T.); 3 Sports School, Autonomous University of Baja California, Ensenada 22890, Mexico; 4 Faculty of Physical Activity Sciences, Autonomous University of Chihuahua, Chihuahua 32310, Mexico; 5 Institute of Biology, National Autonomous University of Mexico, Mexico City 04510, Mexico; miguel.murguia@ib.unam.mx; 6 Nursing Department, Faculty of Health Sciences, University of Alicante, San Vicente del Raspeig, 03690 Alicante, Spain; josemiguel.ms@ua.es; 7 Medical and Psychology School, Autonomous University of Baja California, Tijuana 22390, Mexico

**Keywords:** anthropometry, biomechanics, older adults, nutritional status, physical anthropology

## Abstract

This study investigates the immediate effects of acute postural exercises on the stature of seniors, focusing on changes in both upright and supine stature measurements. A within-subject design with repeated measures was applied, involving seniors participating in continuous exercises aimed at enhancing core musculature strength and promoting muscle relaxation. Stature measurements were recorded pre- and post-exercise in both upright and supine positions, alongside assessments of body mass index (BMI) category classifications. The results revealed a post-exercise increase in stature ranging from 0.9 to 6.0 cm and from 0.2 to 7.2 cm in upright and supine positions, respectively, with an average increase of approximately 3.5 cm in both upright and supine positions. Statistically significant and clinically relevant changes were observed (*p* < 0.05), including a modification of BMI by approximately two units, reclassifying 55% of participants from overweight or obese to normal weight or overweight. Furthermore, the similarity between post-exercise upright stature and pre-exercise supine stature suggested that the supine position might provide a more accurate measure of stature in seniors. Conclusively, acute postural exercises have an immediate positive impact on the stature of seniors, suggesting their potential utility in clinical settings for accurate stature measurement. However, BMI results should be interpreted with caution because they are only related to the acute change in stature and therefore may lead to the misinterpretation of the study findings, so future studies focused on evaluating the chronic effect of postural exercises integration on the health outcomes of older adults are needed to demonstrate their potential utility in clinical settings to improve postural health and general well-being.

## 1. Introduction

Stature is the perpendicular distance between the transverse planes of the vertex and the inferior aspect of the distance from the feet to the highest part of the head [1]. The maximum stature is genetically determined by inheritance [2] and modified by environmental, nutritional, and socioeconomic factors [3]. Stature is a multidisciplinary and multifaceted study parameter encompassing evolutionary, anthropological, political, cultural, social, economic, and public health aspects. It predicts social behaviors, chronic diseases, and, together with body mass, can predict one of the main health status indices used in epidemiological studies, the body mass index (BMI) [4,5,6].

Anthropological, socioeconomic, and health studies consider the maximum stature achieved as a parameter to determine, in a secular way or within certain time–space, the state of the nutritional health (malnutrition and growth delay) of the individual and populations [4,5]. So, stature is a public health parameter that aids in the understanding of social behaviors and health problems. Stature is affected by posture, joint flexions, spine curvature, body weight, malnutrition, anemia, reduced functional capacity, and poor health status, and it is influenced by age and gender [7,8,9].

Moreover, posture, including upright height and normal spine curvature, is affected by fatigue, muscle contractions, muscle tone, and the force at the trunk level, where systematic physical activity improves posture [8,10,11,12]. It has been shown that regular physical exercise improves posture without increasing stature in well-fed young people, e.g., Wang et al. [9] reported that eight weeks of specific strength and stability exercises for the trunk decreased chronic low back pain and improved posture in young adults; they do not report effects on height. Also, there exist in the literature reports that rehabilitation exercises reduce pain and improve postural balance and the quality of life of women with postmenopausal osteoporosis with vertebral fragility fractures [13].

Like any evaluation of health status, an incorrect measurement of stature will make the precise application of preventive or therapy treatments difficult, especially for calculating energy/calories needed for dietary prescription, physical exercise programs, and lifestyle modifications [14,15]. However, the accurate measurement of maximum stature is often hindered by technical limitations, postural difficulties, diseases, musculoskeletal injuries, and aging, rendering it problematic in clinical practice and research [16]. Above all, stature measurement is crucial for understanding malnutrition prevalence among the elderly (9.1% to 12.6%), particularly in low-income countries [17].

The aging process includes often accompanied by physiological changes, including alterations in posture and stature [4,18]. Also, when maximum stature is reached, it remains unchanged until age 40, when it decreases gradually with quadratic kinetics. These changes can impact seniors’ quality of life, mobility, and overall health [4]. The typical postural changes observed in the elderly, such as increase kyphosis, forward head posture, and decreased spinal extension, are not merely cosmetic but associated with risks of falls, reduced respiratory efficiency, and diminished functional capacity [18,19]. Because of this, understanding the immediate effects of acute postural exercise on senior stature is critical for developing effective strategies to mitigate risk related to postural changes.

Studies suggest that older adults improve postural control through various exercises and training programs despite having inherent disadvantages in postural stability compared to younger adults [20]. Moreover, it has been shown in various research that older adults face several challenges in incorporating physical activity into their lives. These are problems that make them socially more vulnerable, affecting their medical treatments and fitness health [21]. To our knowledge, there are no works that evaluate acute stature modifications (standing and supine) due to posture-improving treatments in older adults. Therefore, we hypothesize that acute postural exercises will improve older adults’ stature alignment (standing and supine). The main objective of our research is to explore the acute effects of postural exercises on the stature of the elderly.

## 2. Materials and Methods

### 2.1. Study Design

A one-group pretest–post-test experimental design was conducted to determine the effects of acute postural exercises on the stature of seniors. The protocol and procedures were approved by the Autonomous University of Baja California (UABC) bioethics committee (998-/2020-1; Supplementary File S1). Moreover, the World Medical Association’s Declaration of Helsinki guidelines were followed [22]. In addition, the study design, as well as the development of the manuscript, followed the STROBE statement [23].

### 2.2. Participants Eligibility

The inclusion criteria for the participants were (i) age ranging between 60 and 90 y, (ii) having good physical and mental health verified by clinical anamnesis by a health professional, (iii) and being a Mexican descendant. The exclusion criteria were (i) reporting a current musculoskeletal injury, (ii) having a poor postural alignment according to the New York Posture Rating [24], and (iii) presenting a pain or physical incommodity at the moment of the performance of any of the postural exercises. The minimum sample size calculated (jpower, Jamovi v. 2.4) was of 44 to reliably (with probability greater than 0.9) detect an effect size of δ ≥ 0.5, assuming a two-sided criterion in a paired sample *t*-test for detection that allows for a maximum type I error rate of α = 0.05 to be obtained. Participants were informed of the study rationale as well as all its procedures. Their inclusion was formalized by signing informed consent (Supplementary File S2), anonymity and confidentiality were carefully enforced, and agreeing that all the participant’s data will be stored for five years after the study’s completion.

### 2.3. Procedure

First, an open invitation was posted on the university’s official site, and after receiving the first participants, snowball sampling was used, resulting in reaching more than double the minimum sample size needed. All the participants were informed of the characteristics of the study, and their collaboration was requested. Once they agreed to participate, they signed the informed consent form, passed it to the basal evaluation, developed the postural exercises, and completed the final evaluations on the same day. Stature measurements were taken with the participant in a standing position and then in the dorsal decubitus position. 

#### Postural Exercises

**Figure 1:**
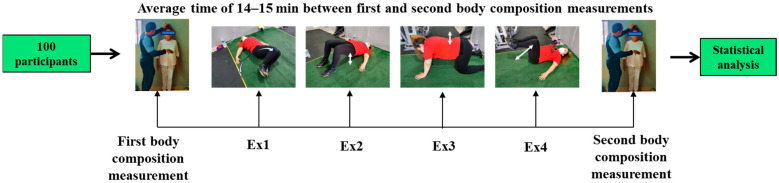
Order of continuous exercises applied for posture correction. Ex1: left–right hip rotation, Ex2: hip elevation–depression, Ex3: spine elevation–depression, Ex4: hip flexion–extension.

The postural correction exercises [10] were performed at the Conahcyt National Laboratory of Body Composition and Energetic Metabolism (LanCoCoME) in Mexico and were supervised by a physical exercise professor and consisted of two sets of four different exercises, including left–right hip rotation, hip elevation–depression, elevation–depression of the supine, and hip flexion–extension (Figure 1). The specific position for each exercise, execution time, sets, and repetitions are described in Table 1. During the continuous exercises, participants performed isometric and eccentric contractions of the trunk muscles, with deep inspirations during isometric contractions and expiration during the eccentric contractions, with 15 s of rest between sets and between exercises. Stature measurements were taken immediately before and after the postural exercises.

### 2.4. Anthropometric Measurements

Kinanthropometric measurements were taken according to the ISO 7250-1:2017 [25] and the International Society for the Advancement of Kinanthropometry (ISAK) standard [1] by certified ISAK anthropometrists supervised by an instructor trained to level 3. Weight was recorded on a horizontal surface with a pre-calibrated Tanita digital scale (BC-418, Tanita Corporation, Tokyo, Japan), showing a sensitivity of 0.1 kg after the digital sensor had stabilized. Stature was recorded visually using a portable stadiometer (SmartMet^®^, Guadalajara, Mexico) with a sensitivity of 1 mm and attached to a flat wall. Stature was measured while the participant stood erect and then supinated, with their body evenly distributed on both sides, or feet and heels together as close as possible, legs and trunk straight without stiffness, and the head erect and in the Frankfort plane. The arms hung relaxed with the palms of the hands beside the body. A stadiometer was used to measure the vertical distance between the standing surface and the top of the head (vertex) at the end of inhalation. All kinanthropometric measurements were measured two or three times depending on whether the technical error of measurement (TEM) between the first two measurements was greater than 1% for the measurements, taking the mean or median, respectively, for subsequent analysis.

### 2.5. Statistical Analysis

The equality of variances and normal distribution of the data were analyzed by Levene’s and Kolmogorv–Smirnov tests, respectively. The differences before and after exercise were analyzed by a dependent samples *t*-test. Because men are, on average, taller than women, and height decreases with age, these two variables were incorporated into the statistical analysis. The possible influence of gender and age was investigated with mean differences by covariance (ANCOVA) analysis. The effect size was determined by Cohen’s d, and statistical significance was set at a *p*-value < 0.05. Analyses were performed with the statistical software Jamovi version 2.4.

## 3. Results

In total, 100 older adults of both genders [50 women: 67.7 ± 5.9 years (range = 60–86 years), BMI = 26.3 ± 1.9 kg/m^2^ (range = 21–30.7 kg/m^2^); 50 men: 68.1 ± 5.8 years (range = 60–83 years), BMI = 26.2 ± 2.0 kg/m^2^ (range = 20.9–30.3 kg/m^2^)] were evaluated. Stature, measured both standing and supine, increased significantly (*p* < 0.05) by ~3.5 cm following posture correction exercises; conversely, BMI decreased (*p* < 0.05) by ~one unit (Table 2). The effect size of the differences was large (Cohen’s d > 0.8).

**Figure 2:**
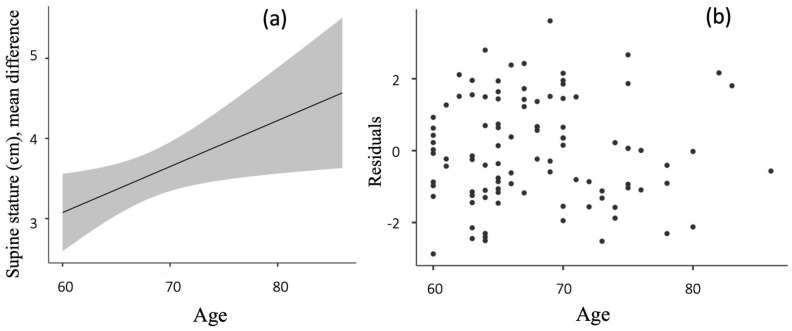
Effect of postural exercises on the supine stature correction. (**a**) Regression line; (**b**) measurement errors of the regression model.

Age was positively associated with post-exercise supine stature increase. The linear prediction equation result of the present study was as follows: Y = 0.057x − 0.371; (R^2^ = 0.052; *p* = 0.023), where Y is the stature increase (cm) and x is age (years), and the rest of the regression model coefficients values are depicted in Table 3. Older age is a great predictor of the greater effect of postural exercises on the supine stature (Figure 2a), with errors of the regression model being distributed randomly (Figure 2b). The sex category did not influence the results.

## 4. Discussion

This study provides compelling evidence that acute postural exercises can impact older adults’ stature, as reflected in the immediate increase observed post-exercise. The acute increase in stature, without body weight changes, even affects the effective reduction in BMI, with both stature and BMI being statistically significant and clinically relevant. These changes are noteworthy as they suggest that postural exercises can quickly alter physical parameters often considered to be stable in the senior population. The acute postural exercises demonstrated in this study specifically target the core musculature, contributing to an increased upright stature. By strengthening the core, these exercises help maintain spinal alignment and reduce curvature, particularly in the lumbar and thoracic regions. This is consistent with the findings obtained by Katzman et al. [26], who observed significant improvements in kyphotic posture following targeted postural exercises in older adults. Moreover, this work observed that maximum stature is influenced by posture, fatigue, muscle tone, and age in a range from 0.9 to 6.0 cm and from 0.2 to 7.2 cm in upright and supine positions, respectively. As expected, postural exercises similarly affect upright and supine stature (~3.5 cm). Since we did not assess the possible existence of muscle contractures, which undoubtedly affect posture and stature [8,27], we consider that the postural exercises had two effects: The first was that they increased the muscle tone of the core musculature, decreasing the curvature of the spine, an effect which was mainly observed during the erect position. The second was that muscle relaxation was favored, an effect observed during the supine position. In both cases, they increased stature. Our results are not only statistically significant (*p* = 0.001) and have a high effect size (Cohen’s d = 2.4) but they are also clinically relevant, as BMI is modified by ~one unit (95% CI: −1.1–−0.9); moreover, 55% of participants were no longer assessed as overweight or obese but rather as normal weight or overweight.

However, BMI is determined by a simple equation that is calculated using body mass (kg) divided by stature (squared meters) (BMI = body mass (kg)/[stature (m)]^2^), but it should be interpreted with caution because our results on BMI modifications are not related to changes in body composition and are only related to the acute change in stature; therefore, they may lead to misinterpretation of the study findings. Therefore, to avoid the methodological limitations of BMI calculation, additional longitudinal studies evaluating body compartments (fat, muscle, lean, or bone mass) are needed that may be directly related with pathological conditions or appropriate medical treatments in the elderly [28].

Comparing our findings with similar recent studies reveals both consistencies and variations. For instance, a study by Katzman et al. [29] corroborates our observation that postural exercises can significantly enhance upright posture in seniors. However, our study extends these findings by quantifying the exact increase in stature, providing a novel insight into the measurable impacts of such exercises. Several works report that postural training programs, such as muscle strengthening, spinal extension, and balance exercises, improve posture in seniors [26,30]; these exercises allow for the maintenance of a more upright stance, reduce forward head posture, and decrease kyphotic curvature. Furthermore, this work demonstrates that a series of postural exercises in the core immediately increases stature among seniors of both sexes; therefore, there is a need to implement this type of postural exercise before taking any height measurement. The similarity between the measurement of stature in the upright position after the exercises and stature in the supine position before exercises indicates that the supine position is a better measure of stature, as the force of gravity or muscle weakness does not influence it.

While all participants in the present study were healthy individuals, a weakness of this study was the failure to assess strength, muscle mass, and joint flexibility, which are affected by age and a sedentary lifestyle [5,10]. This means that with increasing age and a sedentary lifestyle, the influence of gravity on posture and stature is more remarkable, in our case affecting maximum stature, at a rate of approximately 0.1 cm per year or 1 cm per decade. Cross-sectional studies have predicted rates of stature decrease of 0.14 cm/year in men and 0.21 cm/year in women, i.e., approximately 5.5 cm in men and 8 cm in women over a lifetime [31]. In contrast, longitudinal studies of 4–8 years have shown decreases in stature of 2–4 cm between the ages of 50 and 90 [4]. In as little as a decade or a couple of generations, stature is likely to reflect socioeconomic status and food availability [6].

The relationship between age and the increase in post-exercise supine stature, here found, is particularly interesting. The positive correlation implies that older individuals may benefit more from these exercises regarding stature enhancement. This finding is crucial as it indicates the potential of postural exercises to counteract age-related posture deterioration, a common issue in geriatric health. Furthermore, the finding that the supine stature post-exercise closely matches the pre-exercise upright stature suggests that supine measurements might be a more accurate reflection of an individual’s true stature. This insight could have relevant implications for clinical practice, particularly in accurate stature and quality of life assessments in the senior population. Age negatively affects posture and stature [4], mainly due to the influence of gravitational force, weakness, and loss of muscle mass, increasing joint curvatures [12]. In the supine position, controlling in some way for the influence of these two factors, we verified the inverse relationship reported between stature in the supine position and age (R^2^ = 0.052; *p* = 0.042). This inverse relationship is confirmed by the characteristics of the upright posture, as described below: the human body is a flexible, multi-jointed structure, adaptable to different loads and stresses, generating posture and movement through ligaments, tendons, and muscles. The primary function of the ligament is to stabilize the joint by preventing abnormal movements and, in turn, generate force through the tendons, allowing for movement and the development of mechanical work. Due to gravitational force and its effects on all bodies, in our case on joints in the body, the higher the degree of inclination, the greater the transverse load (also called torsion or moment) on the joints.

In the spine, being a multi-joint system, load is distributed in all joints, but especially in the area with the greatest arch (lumbar area), where the greater the separation from the vertical axis of the body structure, the greater the load on the joints and the greater the muscle fatigue. Therefore, weak musculature, caused mainly by a sedentary lifestyle, increases fatigue and prevents correct standing posture. By another size, postural exercise improves proprioception [32] and increases the activation of postural muscles [33], which are crucial for maintaining an upright posture.

The positive outcomes observed in this study have relevant public health implications, particularly in the context of an aging population. Improving posture and reducing fall risks through such exercises can contribute to healthier aging and potentially reduce healthcare costs associated with fall-related injuries. As Tinetti and Kumar [34] indicated, fall prevention strategies are crucial in reducing both the direct and indirect costs associated with aging populations.

While this study highlights the immediate benefits of postural exercises, the long-term sustainability of these improvements remains to be explored. It would be beneficial for future research to investigate how ongoing exercise regimens impact posture and stature over extended periods. This is critical, as noted by Daly et al. [35], in understanding the long-term benefits of physical interventions in the elderly. Considering these findings, healthcare providers are encouraged to integrate postural exercises into the routine care of senior patients. As everyone’s health status varies, exercises should be tailored to accommodate different levels of mobility and health conditions. This approach aligns with recommendations made by Daly et al. [35], emphasizing the need for personalized exercise programs in elderly care.

### 4.1. Study Limitations

Even though the present study shows evidence of the immediate effects of acute postural exercises on the stature of the elderly, some limitations exist, including the following: (i) sample size, due to it being obtained by convenience, so including a larger sample size in future studies will result in more representative results; (ii) there were no restrictions to daily physical activity or body mass, so the overall results may not translate to other populations of older adults; (iii) it must be considered that BMI is not a reliable indicator of health because it is not directly related to body fat, so more studies are needed focusing on evaluating specific body composition parameters related to the health of older people, such as body fat, muscle skeletal mass, and bone density; (iv) we did not apply a control condition, so we cannot confirm that only resting in supine position would not have similar results in the elderly stature; and (v) another limitation is that the study results are not representative of a possible real situation because we cannot confirm that the stature increase found during the study period was due to only acute effects of the exercises, so (vi) longitudinal studies focused on evaluating the chronic effect of postural exercises integration on the health outcomes of older adults are needed to demonstrate their potential utility in clinical settings to improve postural health and general well-being.

### 4.2. Practical Implications

- Accurate stature measurement is crucial for the proper diagnosis and treatment of older adults. This is especially important in dietary and nutritional studies.
- Understanding the gradual decline in stature over time can prevent future postural pathologies.
- Considering the degree of height decrease as a clinical variable in the patient’s medical is recommended.

## 5. Conclusions

Acute postural and flexibility exercises on the core enhance the stature of seniors and standardize stature measurements, thereby contributing to improved physical health and functional independence. Stature measured in the supine position is like that measured in the upright position after exercises. The stature measured in the supine position and after postural and flexibility exercises is the maximum stature found, which tells us that it is the closest to the stature reached during adolescence. These results reaffirm the value of acute postural exercises in enhancing the stature of seniors and offer a practical, non-invasive method to immediately improve posture, which is a key component of older adults’ health and quality of life. This work suggests that while the benefits are immediately noticeable, the changes in BMI are not related to body composition changes due to not taking into account which body compartment that body mass refers to, so consistent practice is essential for long-term postural health. It calls for further research into how these immediate changes can translate into sustained postural improvements, over time evaluating body compartments to avoid misclassifying people due to changes in their body composition that occur in the aging process (increase in fat mass and decrease in muscle mass).

## Figures and Tables

**Table 1:** Training load and execution of posture correction exercises.

Position and Order of the Exercises	Exercises	Execution Time	Sets and Rest Time	Reps
1. Dorsal decubitus	Left–right hip rotation	4 s during each repetition	2 sets, 15 s rest between sets	10–12
2. Dorsal decubitus	Hip elevation–depression	10 s in suspension	2 sets, 15 s rest between sets	10–12
3. Ventral decubitus in 4 points	Elevation–depression of the spine	8 s for each repetition	2 sets, 15 s rest between sets	10–12
4. Dorsal decubitus	Hip flexion–extension	4 s in each repetition	2 sets, 15 s rest between sets	10–12

**Table 2:** Acute effect of posture correction exercises on stature and BMI.

Variable	Initial	Final	Mean Difference ± SEM (95% CI)	*p*-Value	Effect Size (Cohen’s d)
Standing stature (cm)	167.1 ± 8.1	170.6 ± 7.9	3.45 ± 0.12 (3.19–3.71)	<0.001	2.65
Supine stature (cm)	170.9 ± 7.5	174.4 ± 7.4	3.53 ± 0.15 (3.23–3.82)	<0.001	2.39
BMI, kg/m^2^ (standing)	26.3 ± 1.9	25.2 ± 1.9	−1.05 ± 0.04 (−1.14–−0.98)	<0.001	−2.57
BMI, kg/m^2^ (supine)	25.1 ± 1.8	24.1 ± 1.8	−1.00 ± 0.04 (−1.09–−0.92)	<0.001	−2.34

Paired samples *t*-test was used to calculate *p*-values. SEM: standard error of the mean; CI: confidence interval; BMI: body mass index.

**Table 3:** Regression model coefficients on supine stature.

Predictor	Estimate	SEM	*t*	*p*-Value	R²	Adjusted R²
Stature						
Intercept	−0.3713	1.6922	−0.219	0.827	0.0518	0.0421
Age, years	0.0574	0.0248	2.313	0.023		

Delta values: final vs. initial differences. SEM: standard error of the mean.

## Data Availability

Any researcher that contacts the corresponding author, M.A.H.-L. (marco.antonio.hernandez.lepe@uabc.edu.mx), will have access to the study data, following the informed consent provided by participants on the use of confidential data.

## Supplementary Materials

The following supporting information can be downloaded at: https://www.mdpi.com/article/10.3390/sports12030085/s1, Supplementary File S1: UABC bioethics committee approbation letter; Supplementary File S2: Informed consent letter.
