# Supplementary material for: Enhanced Stature in the Elderly: The Immediate Impact of Acute Postural Exercises
Source: Sports (Basel). 2024 Mar 20;12(3):85. doi: 10.3390/sports12030085 (PMC10974903; doi:10.3390/sports12030085)
Supplement: Supplementary file 1 [file sports-12-00085-s001.zip › Supplementary File S1.pdf]

UNIVERSIDAD AUTÓNOMA DE BAJA CALIFORNIA

FACULTAD DE MEDICINA Y PSICOLOGÍA

Tijuana, B.C., a 30 de noviembre del 2020
Oficio, No. 998/2020-2

DR. MARCO ANTONIO HERNÁNDEZ LEPE
PRESENTE.-

Por medio de este conducto, me permito informarle que se sometió a revisión a la Comisión de Bioética de esta Facultad, la solicitud de revisión del proyecto de investigación titulado: **“EFFECT OF A SYSTEMATIC PHYSICAL EXERCISE PROGRAM AND SPIRULINA MAXIMA SUPPLEMENTATION ON THE BODY COMPOSITION, PHYSICAL FUNCTION AND BLOOD LIPID PROFILE IN SEDENTARY OLDER ADULTS WITH EXCESS WEIGHT: A DOUBLE-BLIND, RANDOMIZED, CROSSOVER, PLACEBO CONTROLLED TRIAL”**, el cual tuvo como resultado el siguiente dictamen: **APROBADO** (se anexa).

Sin más por el momento, me despido de usted enviándole un cordial saludo.

ATENTAMENTE
¨POR LA REALIZACIÓN PLENA DEL HOMBRE¨

DRA. JULIETA YADIRA ISLAS LIMÓN
DIRECTORA

UNIVERSIDAD AUTÓNOMA DE BAJA CALIFORNIA
FACULTAD DE MEDICINA Y PSICOLOGÍA
CAMPUS TIJUANA

C.c.p.- Dra. Ana Laura Martínez Martínez.- Coord. Inves y Posgrado FMP
C.c.p.- Dirección
