# Supplementary material for: Enhanced Stature in the Elderly: The Immediate Impact of Acute Postural Exercises
Source: Sports (Basel). 2024 Mar 20;12(3):85. doi: 10.3390/sports12030085 (PMC10974903; doi:10.3390/sports12030085)
Supplement: Supplementary file 1 [file sports-12-00085-s001.zip › Supplementary File S2.pdf]

CONSENTIMIENTO INFORMADO

Nombre del participante: ____

Se te está invitando a para participar en el estudio denominado **Efecto de un programa de ejercicio físico sistemático y la suplementación con *Spirulina maxima* sobre la composición corporal, función física y perfil de lípidos sanguíneos en adultos mayores sedentarios con sobrepeso**. El consentimiento informado es un requerimiento ético en cualquier protocolo experimental. Siéntete con absoluta libertad de preguntar sobre cualquier aspecto que te ayude a aclarar todas tus dudas. Antes de decidir si participas o no, debes conocer y comprender cada uno de los apartados que adelante se describen de forma escrita y te son explicados por el personal del estudio.

I. Justificación y objetivo del estudio

La población de adultos mayores a nivel mundial se encuentra en un crecimiento mayor que cualquier otro grupo de edad, lo cual puede ser explicado por la disminución existente en la tasa de natalidad y el incremento de la esperanza de vida. En lo que respecta a México, también existe un aumento demográfico de adultos mayores, de acuerdo al perfil epidemiológico, en el año 2000 las personas mayores de 60 años representaban el 6.8% del total de la población, y se prevé que para el año 2050 esta cifra supere el 28% del total.

La expectativa de vida saludable de adultos mayores en México es de 65.8 años, y la expectativa de vida de la población general es de 74.4 años, lo que significa que la población que llega a esta edad se encuentra en un alto riesgo de desarrollar alguna enfermedad o dependencia durante aproximadamente nueve años de su vida. Para la población mayor de 60 años, las tres más frecuentes condiciones de salud reportadas fueron hipertensión (40%), diabetes (24.3%) e hipercolesterolemia (20.4%). Estas enfermedades afectan todos los sistemas del cuerpo e impiden llevar a cabo actividades de vida cotidianas como caminar, comer, preparar o comprar alimentos, bañarse, entre otras. En este sentido, es necesaria la

25-11-2020 / 25-11-2022

Facultad de Medicina y Psicología
MEDICINA
Comité de Bioética

búsqueda de un estilo de vida saludable, en el que se incluye una alimentación saludable que incluya suplementación con nutraceúticos y la práctica diaria de actividad física.

Respecto a la suplementación con nutracéuticos, la cianobacteria *Spirulina maxima* (*S. maxima*) es una fuente importante de proteínas, carotenoides, compuestos fenólicos y ácidos grasos poliinsaturados, a la cual se le han comprobado diversas actividades biológicas como efectos hipolipemiantes, sin embargo, los estudios existentes de efectos de *S. maxima* sobre la composición corporal, la función física y el perfil de lípidos sanguíneo en humanos, principalmente en adultos mayores, tienen un bajo nivel de evidencia y ausencia de diseños experimentales adecuados, por lo que no se puede asegurar su valor como nutracéutico.

Los efectos de la realización de ejercicio físico sistemático en adultos mayores ha mostrado tener efectos positivos en la composición corporal y el perfil de lípidos sanguíneo, por lo que en el presente estudio se plantea evaluar la sinergia que presenta un suplemento como la *S. maxima* en un plan de ejercicio físico sistemático sobre la composición corporal, la función física y el perfil de lípidos sanguíneos de adultos mayores sedentarios con sobrepeso en un estudio clínico aleatorizado, doble ciego, cruzado con placebo controlado.

Características del estudio

Los sujetos serán citados en el laboratorio de composición corporal de la Universidad Autónoma de Baja California (UABC) en condiciones de ayuno de 8 a 10 h para realizarles los siguientes procedimientos y evaluaciones:

1. Para conocer el estado de salud general se les realizará un examen médico-clínico con estudios antropométricos y de laboratorio (química sanguínea y perfil de lípidos).

2. Para conocer el efecto de los tratamientos sobre la composición corporal se realizarán mediciones antropométricas por el método estandarizado por la Sociedad Internacional para la Estandarización Antropométrica (ISAK, por sus siglas en inglés), además de una medición de densitometría corporal por pletismografía (BodPod, USA).

3. Para conocer el efecto de los tratamientos sobre el acondicionamiento físico general (AFG) se realizará el “Senior Fitness Test”. La fuerza de los miembros inferiores (SFT-LBS) se evaluará mediante el test de 30 s de levantarse y sentarse en una silla. La fuerza de los miembros superiores se valuará por curl de brazo con mancuerna durante 30 s (hombre= 8 lb,

Facultad de Medicina y Psicología
MEDICINA
Comité de Bioética

mujer= 5 lb). La flexibilidad de los miembros inferiores se evaluará por la prueba de flexión en silla, donde los participantes flexionan lentamente las articulaciones de la cadera, hasta alcanzar la maxima extensión posible, incluso puede llegar a pasarse la punta de los dedos del pie. La flexibilidad de los miembros superiores se evaluará por el test de las manos por detrás de la espalda, donde la distancia entre las puntas de los dedos medios se medirá independientemente del alineamiento de la espada. La agilidad se evaluará por el test de 2.44 m, donde los participantes deben levantarse de una silla, caminar lo más rápido posible 2.44 m y sentarse nuevamente. La Resistencia aeróbica se evaluará por el test de 2 m de marcha de pie en el mismo sitio, donde los participantes deben realizar la mayor cantidad de repeticiones posibles.

4. Para conocer el efecto de los tratamientos y de las modificaciones en el AFG sobre los parámetros bioquímicos arriba señalados, se tomarán, en tubos con anticoagulante, 10 mL de sangre venosa en su primer visita al laboratorio, así como a las 12, 14 y 26 semanas después (cuatro tomas en total). El plasma sanguíneo se separará por centrifugación refrigerada a 2500 rpm, a 4°C durante 20 minutos y se congelará a -80C hasta su análisis conjunto.

5. Los participantes que pertenezcan al grupo que realizará ejercicio físico sistemático, este será 5 días a la semana, donde recibirán las rutinas de ejercicio bajo la supervisión técnica de un investigador certificado en entrenamiento personalizado, con sesiones diarias de 50-70 min a intensidades entre 40-70% de la frecuencia cardiaca de reserva de cada sujeto de estudio; además todos recibirán una intervención nutricional para establecer recomendaciones dietarias individualizadas.

6. Durante todos los procedimientos serás supervisado continuamente por el personal que te atiende mediante la medición de tus signos vitales, todo lo anterior en apego a las normas de salud vigentes (NOM-012-SSA3-2012), incluyendo la toma y manejo (NOM-007-SSA3-2011) y disposición de componentes sanguíneos (NOM-253-SSA1-2012) y material de toma de muestra (NOM-051-SSA1-1993), que será realizado por personal capacitado para atender cualquier incidente en el manejo de muestras biológicas. Por ultimo, en las actividades a realizar deberás utilizar ropa deportiva para evitar la incomodidad durante la ejecución de las actividades y los resultados sean confiables.

Facultad de Medicina y Psicología
MEDICINA
Comité de Bioética

II. Riesgos asociados al estudio

Por la naturaleza del estudio NO EXISTE ningún riesgo o perjuicio en la salud, siempre y cuando te encuentres sano, y si así nos lo manifiestan tanto los exámenes previos arriba señalados como tú durante esta entrevista. Sin embargo, en casos aislados, las pruebas de ejercicio a intensidad máxima podrían ocasionar desmayo, calambres, vómito, lesiones de primer, segundo y tercer grado u otros malestares, sobre todo si las personas previamente traen alguna patología no conocida o no reportada, y que no están acostumbradas a realizar ejercicio intenso. En tal caso, el personal que te atiende tiene entrenamiento en primeros auxilios y te ayudará y canalizará a los servicios de emergencia correspondientes.

III. Aclaraciones

- Tu decisión de participar en el estudio es completamente voluntaria.
- Durante el estudio puedes solicitar información actualizada sobre el mismo al investigador responsable.
- No habrá ninguna consecuencia desfavorable en caso de no aceptar la invitación.
- Para evitar o disminuir posibles riesgos a su salud, el personal del laboratorio supervisará de manera constante diversos parámetros fisiológicos y signos vitales.
- En caso de que desarrolles algún efecto adverso secundario no previsto tienes derecho a no continuar en el estudio y en caso extremo a una indemnización económica, siempre y cuando los efectos adversos sean a consecuencia del tratamiento al que aceptas participar.
- Si consideras que no hay dudas ni pregunta acerca de tu participación, puedes, si así lo deseas, firmar esta carta de consentimiento.
- La información recabada solo será útil para los fines de la investigación y se garantiza el anonimato de todos los participantes. La confidencialidad de la información recabada seguirá los lineamientos estipulados por el Reglamento de la Ley General de Salud en materia de Investigación para la salud (Artículos 13 y 16), Ley de Información Estadística y Geográfica (Artículos 38 y 42) y el comité de bioética de la UABC.
- Recibirás una copia firmada y fechada de este formulario de consentimiento.

Facultad de Medicina y Psicología MEDICINA Comité de Bioética

IV. Consentimiento

He leído y comprendido la información anterior y mis preguntas han sido respondidas de manera satisfactoria. He sido informado de los efectos positivos y posibles efectos negativos del estudio sobre mi salud. La información que he proporcionado al personal responsable de este estudio ha sido veraz, y convengo en participar en el estudio.

Nombre y firma del participante	/ / Fecha
Nombre y firma del testigo	/ / Fecha

Responsables del estudio: **Dr. Marco Antonio Hernández Lepe**

Yo, Dr. ____ he explicado la naturaleza y los propósitos del estudio. Le he explicado a cerca de los beneficios y posibles perjuicios que implica su participación. He contestado a las preguntas en la medida de lo posible y he preguntado si tiene alguna duda. Acepto que he leído y conozco la normatividad correspondiente para realizar investigaciones con seres humanos y me apego a ellas.

Una vez concluida la sesión de preguntas y respuestas, se procedió a firmar el presente documento.

________________________________ ________________________________

Firma del investigador Fecha

________________________________ ________________________________

Firma del participante Fecha

Facultad de Medicina y Psicología
MEDICINA
Comité de Bioética

Referencias

NOM-007-SSA3-2011, para la organización y funcionamiento de los laboratorios clínicos.

NOM-012-SSA3-2012, Criterios para la ejecución de proyectos de investigación para la salud en seres humanos

NOM-051-SSA1-1993, especificaciones sanitarias de las jeringas estériles desechables de plástico.

NOM-253-SSA1-2012, disposición de sangre humana y componentes con fines terapéuticos.

NOM-253-SSA1-2012, disposición de sangre humana y componentes con fines terapéuticos.
